# Binary Mixtures of Meloxicam and L-Tartaric Acid for Oral Bioavailability Modulation of Pharmaceutical Dosage Forms

**DOI:** 10.3390/jfb15040104

**Published:** 2024-04-16

**Authors:** Cristina Macasoi, Viorica Meltzer, Ioana Stanculescu, Cosmin Romanitan, Elena Pincu

**Affiliations:** 1 Department of Analytical Chemistry and Physical Chemistry, Faculty of Chemistry, University of Bucharest, 4-12 Regina Elisabeta Bd., 030018 Bucharest, Romania; cristinamacasoi@yahoo.com (C.M.); viomel@gw-chimie.math.unibuc.ro (V.M.); istanculescu@nipne.ro (I.S.); 2 Horia Hulubei National Institute for Physics and Nuclear Engineering, IRASM Department, 30 Reactorului Str., 077125 Magurele, Romania; 3 National Institute for Research and Development in Microtechnologies (IMT Bucharest), 126A Erou Iancu Nicolae Street, 72996 Bucharest, Romania; cosmin.romanitan@imt.ro

**Keywords:** meloxicam, L-tartaric acid, mechanosynthesis, FTIR, DSC, FT-Raman, XRD, solubility

## Abstract

Binary mixtures of active pharmaceutical ingredients (API) are researched to improve the oral bioavailability of pharmaceutical dosage forms. The purpose of this study was to obtain mixtures of meloxicam and L-tartaric acid because tartaric acid improves intestinal absorption and meloxicam is more soluble in a weakly basic environment. The mixtures in the 0–1 molar fraction range, obtained from solvent-assisted mechanosynthesis, were investigated by differential scanning calorimetry (DSC), Fourier Transform Infrared (FTIR) spectroscopy, Fourier Transform Raman spectroscopy (FT-Raman), X-ray powder diffraction (XRD) and solubility tests. The physicochemical characteristics of the compounds obtained from DSC data reveal, for the first time, the formation of a co-crystal at meloxicam molar fraction of 0.5. FTIR spectroscopy data show the existence of hydrogen bonds between the co-crystal components meloxicam and L-tartaric acid. FT-Raman spectroscopy was used complementary with FT-IR spectroscopy to analyze the pure APIs and their mixtures, to emphasize the appearance/disappearance and the shifts of the position/intensity of vibrational bands, following the formation of hydrogen-bonded structures or van der Waals interactions, and to especially monitor the crystal lattice vibrations below 400 cm^−1^. The experimental results obtained by X-ray powder diffraction confirmed the formation of the co-crystal by the loss and, respectively, the apparition of peaks from the single components in the co-crystal diffractogram. The solubility tests showed that the co-crystal product has a lower aqueous solubility due to the acidic character of the other component, tartaric acid. However, when the solubility tests were performed in buffer solution of pH 7.4, the solubility of meloxicam from the co-crystal mixture was increased by 57% compared to that of pure meloxicam. In conclusion, the studied API mixtures may be considered potential biomaterials for improved drug release molecular solids.

## 1. Introduction

The solubility of the active pharmaceutical ingredients (API) is the most important factor that influences the bioavailability of drugs. When administering drugs orally, substances with low water solubility have low bioavailability in the body because they are not absorbed in large quantities in the gastrointestinal tract [1,2]. The biological properties of pharmaceutical ingredients could be affected by physical, chemical, and physiological factors such as temperature, pH, O_2_, and enzymatic activity affecting its potential therapeutic activity, as well as chemical instability and high degradability when it is consumed and during its path through the stomach due to its low pH [3]. Because a very large number of active pharmaceutical ingredients have low solubility in aqueous media or in dissolution media with pH characteristic of the gastrointestinal tract, it is very important to obtain pseudopolymorphic forms with improved physical properties. To increase the dissolution rate, a pseudopolymorphic form can be obtained using a very soluble coformer and a poorly soluble active pharmaceutical ingredient. In this way, the poorly soluble drug is trapped in the interstitial space between the conformer molecules, and thus, a faster release of the active pharmaceutical ingredients is obtained.

There are many methods to improve the solubility of these substances, including complexation, the creation of microemulsions, micelles, by mixing in a common solvent and slow evaporation, the formation of liposomes, the formation of polymeric micelles, the formation of salts, the reduction of the particle sizes of substances or co-crystallization.

A new way to improve the solubility of active pharmaceutical substances is the formation of co-crystals, obtained by crystal engineering methods such as: mechanosyhensis, solvent evaporation, hot-melt extrusion, complexation, cryosynthesis, electrodialysis, etc. The co-crystals are crystalline solid compounds, composed of at least two substances in the same crystal lattice. Co-crystals are crystals with multiple components or crystalline complexes stabilized by various types of interactions, including hydrogen bonds or van der Walls forces. Co-crystals exhibit short-range order between neighboring atoms. The same short-range order exists in the eutectic. In both co-crystals and eutectics, this short-range order is associated with the preference for heteromolecular or homomolecular interactions at certain molecular ratios. Pharmaceutical co-crystals can potentiate the physicochemical and mechanical properties of substances, as well as in vivo activity, so they can be used to produce an optimal pharmaceutical formulation [4,5]. In this paper, the binary mixtures of meloxicam (API) and L-tartaric acid (coformer) were obtained by solvent-assisted mechanosythesis and characterized to demonstrate their solubility increase. 

Meloxicam, 4-hydroxy-2-methyl-N-(5-methyl-2-thiazolyl)-2H-1,2-benzothiazine-3-carboxamide-1,1-dioxide is a non-steroidal anti-inflammatory drug (NSAID) that has been used in rheumatoid arthritis treatment, osteoarthritis treatment, ankylosing spondylitis treatment and other diseases for the relief of mild-to-moderate pain [6]. Studies have emphasized that as a relatively selective COX-2 inhibitor has also shown anti-tumor responses against colorectal and ovarian cancer due to the participation of the COX-2 of the progression of these cancers [7].

Meloxicam is a BCS class II (high permeability, low solubility) substance with a limited aqueous solubility of 9.4 μg/L [8]. The solubility of meloxicam in aqueous media is highly dependent on pH, which can be directly correlated to its multiple ionization states. Under physiological conditions at pH 7.4, the predominant structure of meloxicam is the anionic form, while under acidic conditions, additional molecular species may be present (such as cationic form) [9,10]. Due to its low solubility under acidic and neutral conditions, the time to reach the maximum concentration of meloxicam in the human body is typically 4–6 h, while it can take more than 2 h for the drug to reach its therapeutic concentration in humans [11]. Its solubility in water has to be increased in order to be quickly absorbed by the human body and to obtain a good therapeutic response for acute pain relief. Various strategies have been used in the literature to increase the solubility and bioavailability of Meloxicam, such as co-crystallization.

L-tartaric acid (C_4_H_6_O_6_) is a kind of widely sourced, cheap, and non-toxic hydroxycarboxylic acid substance [12], is an organic acid widely used in the food, pharmaceutical, textile and other industries [13]. L-tartaric acid is an important source of antioxidants that protect the body by enhancing immunity and also aids digestion by improving intestinal absorption. L-tartaric acid has a high solubility in aqueous media and can be used as a coformer in obtaining the pseudopolymorphic forms of the active pharmaceutical ingredients. In pharmaceutical applications tartaric acid is used to produce effervescent salt that helps enhance the taste of oral medications. This is used in many different applications due to its properties, such as the following: antioxidant, acidifier, flavor enhancer, stabilizer and sequestering agent.

In the literature, various meloxicam co-crystals have been reported with various carboxylic acids: 1-hydroxy-2-naphthoic acid, glutaric acid, L-malic acid, salicylic acid, fumaric acid, succinic acid, maleic acid, malonic acid, gentisic acid, 4-hydroxybenzoic, acid, adipic acid, (þ)-camphoric acid, glycolic acid, benzoic acid, DL-malic acid, hydro-cinnamic acid, ascorbic acid, acetyl salicylic acid [11,14,15,16,17,18,19,20,21,22,23] and hydrosulphate monohydrate [21]. The physical chemical characterization methods of meloxicam mixtures were as follows: differential scanning calorimetry (DSC), Fourier Transform Infrared spectroscopy (FT-IR), X-ray diffraction (XRD), complemented by solubility, in vitro release and stability studies [6,8]. The obtaining of these co-crystals was highlighted by DSC, FTIR and PXRD and the solubility tests demonstrated the improvement of their solubility.

Although meloxicam co-crystals with carboxylic acids have been reported in the literature, tartaric acid is not among them. Furthermore, an attempt was made to obtain a co-crystal of meloxicam with tartaric acid, but could not be obtained [11]. This demonstrates that the preparation method and the solvent used play an important role in obtaining these compounds [24].

In this paper, we use meloxicam to prepare a new co-crystal with L-tartaric acid by solvent-assisted mechanosynthesis method. The meloxicam–tartaric acid co-crystal obtained was characterized by the following: DSC and TG methods, FTIR and FT-Raman spectroscopy, XRD and the solubility tests showing its potential pharmaceutical forms usage.

## 2. Materials and Methods

### 2.1. Materials

L-tartaric acid 150.087 g/mol (LGC, Teddington, UK, batch G1057978, purity 97.9%), meloxicam 351.40 g/mol (batch 4198, purity 99.8%), methanol 32.04 g/mol (Merck, Darmstadt, Germany, purity 99.9%), sodium hydrogen phosphate (Merck, purity 99.8%), and sodium dihydrogen phosphate (Merck, purity 99.5%) were commercially available and used without further purification. All working solutions for the solubility tests were prepared using a deionized water/buffer solution of pH 7.4. To prepare 0.2 L of the phosphate-buffered solution, mix 19 mL of solution 2 M of sodium phosphate monobasic with 81 mL 2 M of sodium phosphate dibasic and bring to the mark with deionized water. 

### 2.2. Preparation of Binary Mixtures

**Figure 1:**
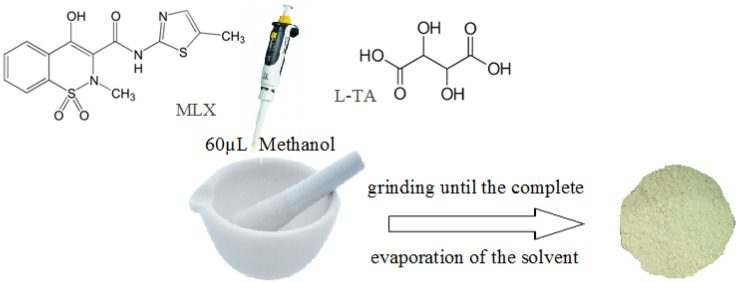
Schematic representation of the co-crystallization of the components by solvent-assisted mechanosynthesis.

The co-crystals were obtained by solvent-assisted mechanosynthesis as follows: the mixtures of a selected mole fraction of meloxicam and tartaric acid were mixed and ground for 5 to 10 min at room temperature using a mortar and pestle to obtain complete homogenization. After that, approximately 0.06 mL methanol was addedadded, and the grinding was continued until the complete evaporation of the solvent (Figure 1). After preparation, the mixtures were kept in a desiccator until they were studied.

### 2.3. Methods of Characterization

The DSC study was performed on meloxicam, L-tartaric acid and their binary mixtures. Their melting temperatures and enthalpies were determined. The determinations were performed with a heating rate of 10 K·min^−1^, a cooling rate of 50 K·min^−1^, over a preset temperature range of (293.15–550.15) K in an inert atmosphere (argon flow rate, 20 mL·min^−1^). The device used, Perkin Elmer Diamond DSC (Waltham, MA, USA), was calibrated with pure indium for temperature and enthalpy. 

The Partner XA balance (Radwag, Radom, Poland) with a weighing accuracy of 10 µg was used to weigh (1–11) mg samples in aluminum crucibles. 

A differential dynamic calorimeter, coupled with a thermogravimeter (STA 409 PC Luxx TG/DSC, Netzsch, Selb, Germany), was used for thermal analysis in nitrogen atmosphere (flow rate, 20 mL·min^−1^), and heating rate of 10 K·min^−1^ in the 300–800 K temperature range. 

Fourier Transform Infrared spectra were recorded in ATR mode using a Perkin Elmer Frontier MIR/FIR spectrophotometer (Waltham, MA, USA) in the wavenumber range (4000–650) cm^−1^ with a resolution of 1 cm^−1^. 

A Bruker Vertex 70 spectrometer (Ettlingen, Germany) coupled to a RAM II module, accessorized with a 1064 nm laser and a liquid nitrogen cooled detector, was used for the registration of FT-Raman spectra at 4 cm^−1^ resolution, in the range (3500–50) cm^−1^ with 64 scans and optimizable laser power of 1 to 500 mW. 

Powder XRD (PXRD) diffractograms were obtained in parallel beam mode, in the angular range 2θ = 5–40°, on a Rigaku SmartLab diffractometer with a 9 kW copper rotating anode source (Osaka, Japan) capable of generating monochromatic X-rays with a wavelength of 0.15406 nm (CuKα1 radiation). XRD diffractograms were recorded at a speed of 3°·min^−1^, using a step of 0.01°·min^−1^. 

The solubility tests were performed for meloxicam and its co-crystal with tartaric acid in deionized water (neutral pH, electrical conductivity of 0.2 μS·cm^−1^) and in a buffer solution of pH 7.4. These were done by the saturation method, using a shaking Erlenmeyer flask for 24 h to reach equilibrium saturation. The tests were performed simultaneously to eliminate the influence of extraneous parameters on the results. The concentration of meloxicam was determined in its saturated solution. After 24 h, a volume of solution was taken from the Erlenmeyer beakers, which was filtered (syringe PVDF filter, diameter—25 mm, individual pore size—0.45 μm, Tisch Scientific) for solid phase removal. The determination of the meloxicam concentration was derived from UV–Vis spectroscopy.

UV–Vis spectra were obtained with the UV–Vis spectrophotometer Perkin Elmer Lambda 45 (Waltham, MA, USA) with a resolution of 1 nm. For each solution, the spectra were obtained in the (200–600) nm range, using a water or buffer solution of pH 7.4 as a blank.

## 3. Results and Discussion

### 3.1. Differential Scanning Calorimetry (DSC) and Thermogravimetry (TG) Analysis

The pure compounds meloxicam (MLX) and L-tartaric acid (TA) and binary mixtures prepared by solvent-assisted mechanosynthesis were studied by DSC and TG in order to establish their thermal stability and phase diagram. 

**Figure 2:**
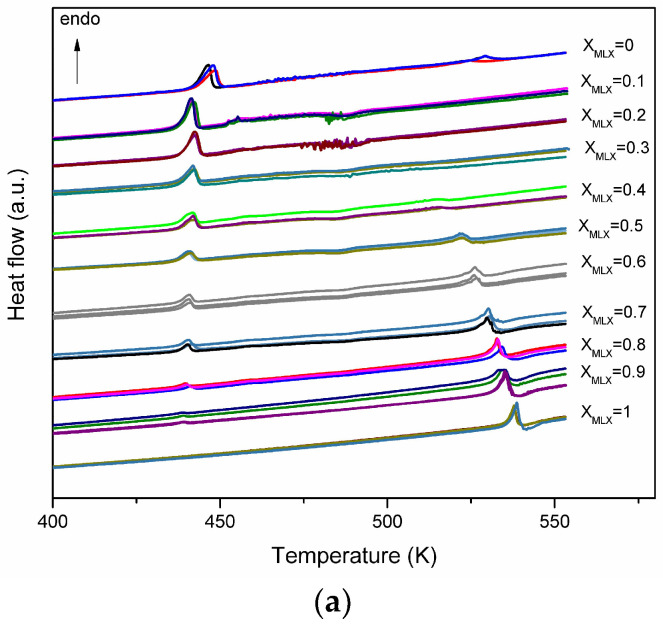

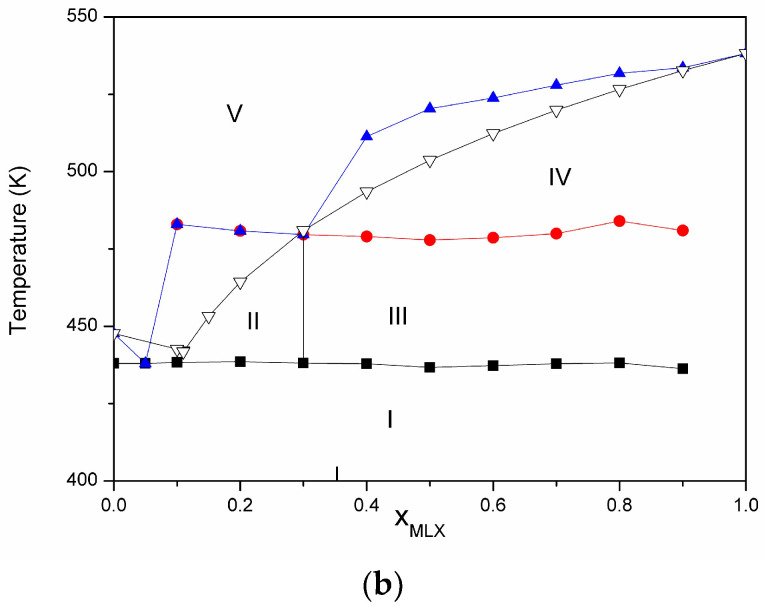
Thermal study of binary mixtures (**a**) DSC curves; (**b**) Phase diagram for binary mixtures meloxicam—tartaric acid: ideal behaviour—empty triangles and real behaviour (eutectic points are shown as filled squares, liquidus points as filled triangles and co-crystal decomposition as filled circles. I–V are the region of coexistence of different phases).

The DSC curves obtained for meloxicam, tartaric acid and binary mixtures with different mole fractions, obtained from the solvent-assisted mechanosynthesis, are shown in Figure 2a. The DSC curve of L-tartaric acid has two endothermic peaks at 447.7 K corresponding to the melting process and at 527.7 K corresponding to the decomposition process. In the case of meloxicam, the DSC curve has one endothermic peak at 538.2 K attributed to the melting process. 

The DSC curves of the binary mixtures of various molar fractions show a first endothermic peak at a temperature of 438 K, a temperature lower than that of the pure compounds, which suggests the formation of a eutectic mixture between components. At a temperature of 479 K, the DSC curves of binary mixtures show an exothermic peak, which can be interpreted as a transition between enantiotropic polymorphic forms of the eutectic; such behavior is relatively uncommon [25] or shows the formation of a compound with an incongruent melting point (decomposition). According to the literature data, a binary mixture is able to form a co-crystal if one or two exothermic processes appear in the DSC curves [26].

For the mixtures with molar fractions between 0.4 and 0.9, the DSC curves show another endothermic peak at temperatures higher than 500 K. This process is due to the melting of meloxicam in excess of the eutectic composition (or co-crystal) and is shifted to lower temperatures than the melting temperature of meloxicam.

The binary phase diagram, temperature–composition plot, for the binary mixture of meloxicam–tartaric acid was performed using the melting and decomposition temperature obtained from the DSC curves, and is presented in Figure 2b. The temperature–composition ideal phase diagram (between the components of the system, there are no interactions and the activity coefficients are equal to unity) is obtained using the liquid transition temperature values determined with the Schröder–van Laar equation [27] as follows:

$$-lnx_{i}=\frac{\Delta^{t}H_{i}^{0}}{R}\left[\frac{1}{T}-\frac{1}{T_{i}^{0}}\right]$$

where: *x_i_* is the mole fraction of the component *i* at the temperature *T*, *R* the gas constant, $\Delta^{fus}H_{i}^{0}$ the molar enthalpy of the fusion of the component *i* and $T_{i}^{0}$ is the melting temperature of the pure component.

If the binary system has an ideal behavior, then the phase diagram is one with a simple eutectic at a composition of the system corresponding to the molar fraction of meloxicam x_MLX_ = 0.11 (Figure 2b—empty triangles).

The binary phase diagram obtained is characteristic of the formation of a co-crystal with an incongruent melting point and has five distinct regions. In region I, there are tartaric acid and meloxicam in the solid phase. In regions II and III, the solid co-crystal and the excess of tartaric acid and meloxicam, respectively, are found. The excess of meloxicam and the decomposition products of the co-crystal are found in region IV.

**Figure 3:**
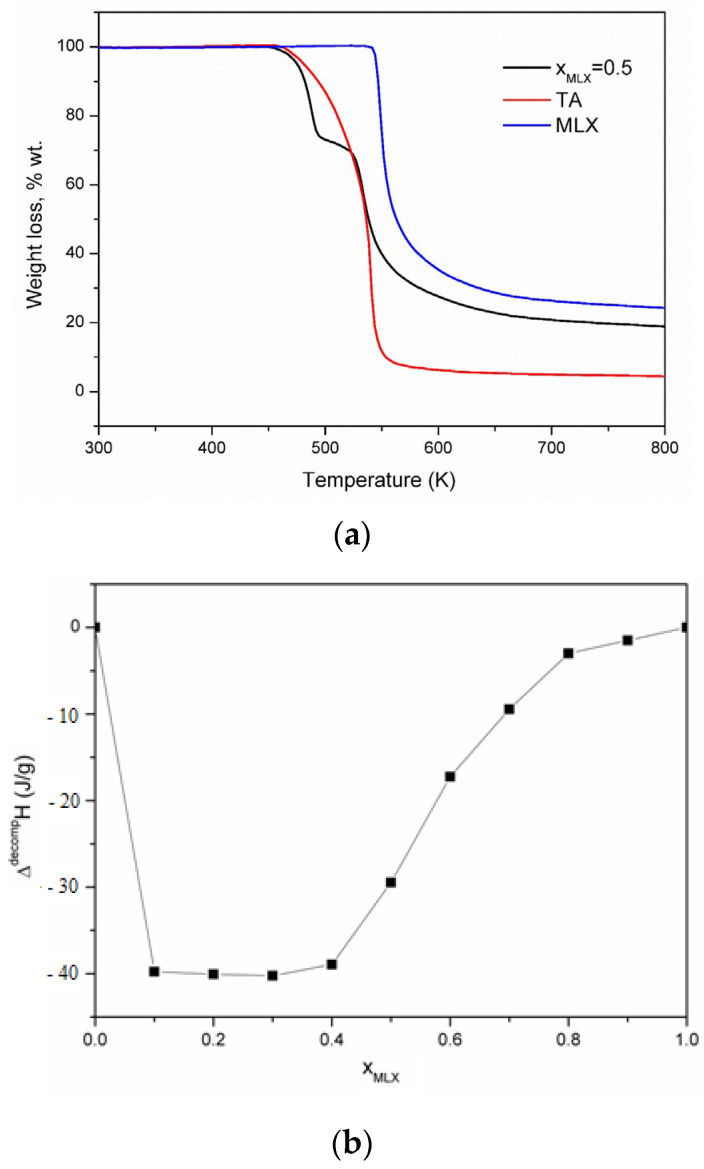
(**a**) TG curves; (**b**) the decomposition enthalpy of the co-crystal.

To see if the exothermic process is a phase transition between two polymorphic forms of the eutectic or a decomposition process of the newly formed compound, the TG curves were recorded and are shown in Figure 3a.

The decomposition temperatures, as well as the mass loss associated with these processes, were determined from the TG curves and are presented in Table 1.

The TG curves for meloxicam (Figure 2b) show that the thermal decomposition process starts at a temperature of 546.65 K, immediately after the melting process, and presents a mass loss of 77%. In the case of tartaric acid, the decomposition process takes place in a single step within a temperature range (458–573) K with a mass loss of 96%.

For the binary mixture, meloxicam–tartaric acid, with molar fraction x_MLX_ = 0.5, the TG curve shows that the exothermic process, which occurs immediately after the melting, is a process of the thermal decomposition of the mixture at a temperature of 474.4 K, with a mass loss of approximately 25%, followed by a second decomposition process at a temperature of 528 K with a mass loss of approximately 57%.

From the TG data, it can be seen that the thermal stability of the mixture decreases in the case of the binary mixture and the thermal decomposition process takes place at temperatures of approximately 474.4 K.

From the DSC curves, the enthalpy of the exothermic process corresponding to the thermal decomposition of the co-crystal was calculated and is represented graphically in Figure 3b. In the range of the molar fractions of meloxicam between 0.1 and 0.3, the enthalpy has approximately the same value, which shows that a co-crystal is formed with a molar ratio between tartaric acid and meloxicam of 3:1. Although the co-crystal is formed at this molar ratio, we chose to further study an equimolecular mixture due to the possible application in the pharmaceutical industry.

### 3.2. FTIR and FT-Raman Study

**Figure 4:**
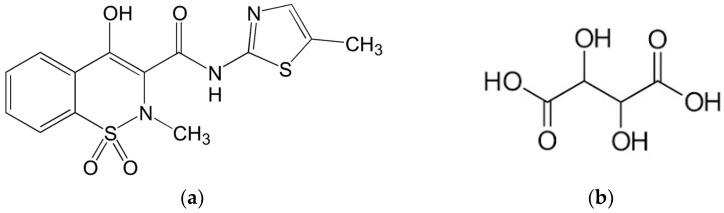
Chemical structure of meloxicam (**a**) and L-tartaric acid ((2R,3R)-2,3-dihydroxybutanedioic acid) (**b**).

FTIR and FT-Raman spectra are used to identify unknown samples by comparison with spectral libraries but also to follow conformational changes due to small perturbations, such as the following: crystalline lattice modification, complexation, host guest interaction, denaturation, aggregation, temperature, pressure and radiation influence, etc. FTIR and FT-Raman vibrational spectroscopy methods are very often used complementarily to detect conformation modification due to molecular interactions by shifts of peak position and intensity. The chemical structure of meloxicam and L-tartaric acid are presented in Figure 4.

**Figure 5:**
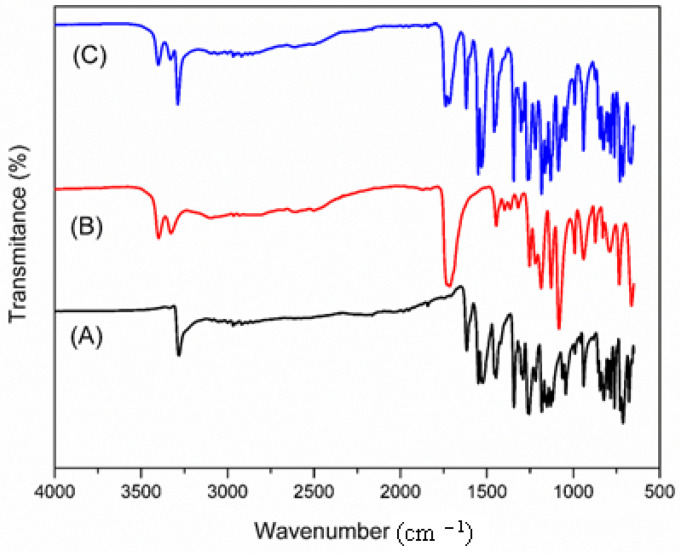
FTIR spectra of meloxicam (A), tartaric acid (B) and their equimolar mixture (C).

The peaks in the FTIR spectra of tartaric acid, meloxicam and their co-crystal are illustrated in Figure 5.

In the FTIR spectrum of meloxicam, the characteristic band of the N-H stretching vibration (secondary amide) can be observed at 3282 cm^−1^, the vibration of C=O the amide group at 1616 cm^−1^, the one associated with the C=N vibration of the thiazole ring at 1523 cm^−1^. The band located at 1262 cm^−1^ can be attributed to the C-N (N-amino nitrogen) stretching vibration, the band from 1119 cm^−1^ can be attributed to the C-O bond (tertiary alcohol) and that from 1043 cm^−1^ can appear due to the S=O stretching vibration of the sulfoxide organic function [28]. On the other hand, in the FTIR spectrum of tartaric acid, the bands at 3399 cm^−1^ and 3328 cm^−1^ correspond with the stretching vibration of the O-H bond of the hydroxyl groups (including those in the structure of the carboxylic groups), and the peaks from 1732 cm^−1^ and 1715 cm^−1^ are assigned to the stretching vibrations of the C=O bond in the carboxylic groups. The spectrum of the co-crystal has all the peaks that the pure substances have, with a slight shift to higher wavenumbers: for meloxicam: 3288 cm^−1^, 1619 cm^−1^, 1530 cm^−1^, 1264 cm^−1^, 1122 cm^−1^ and 1044 cm^−1^, compared to the homologous bands for the pure meloxicam compound located at 3282 cm^−1^, 1616 cm^−1^, 1523 cm^−1^, 1262 cm^−1^, 1119 cm^−1^ and 1043 cm^−1^, respectively; for tartaric acid: 3399 cm^−1^, 3328 cm^−1^, 1732 cm^−1^ and 1715 cm^−1^, compared to the homologous bands for tartaric acid in the pure state located at 3401 cm^−1^, 3330 cm^−1^, 1738 cm^−1^ and 1718 cm^−1^, respectively. These slight shifts in the positions of the vibrational absorption bands of the components in the co-crystal compared to those of the pure compounds suggest the modification of hydrogen bonding interactions between meloxicam and tartaric acid in the co-crystal state.

The FT-Raman technique is used complementary with FTIR spectroscopy for the analysis of pure API and their mixtures to emphasize shifts in the position and intensity of vibrational bands, following the formation of hydrogen-bonded structures or van der Waals (vdW) interactions accompanied or not by the modification of crystal lattices [29,30,31,32].

As previously showed, meloxicam has four prototropic forms [17], and co-crystal formation may be accompanied by structural transitions. According to Pubchem data [33], meloxicam has two hydrogen bond donors (HBD) and seven acceptors, two rotatable bonds, total polar surface area (TPSA) = 136 Å and a lipophilic character (logP = 3), while L-tartaric acid has four HBD centers, six hydrogen bond acceptors (HBA), three rotatable bonds, TPSA = 115 Å and a hydrophilic character (logP = −1.9), indicating the possibility of the formation of various hydrogen and van der Waals bonds when mixed.

**Figure 6:**
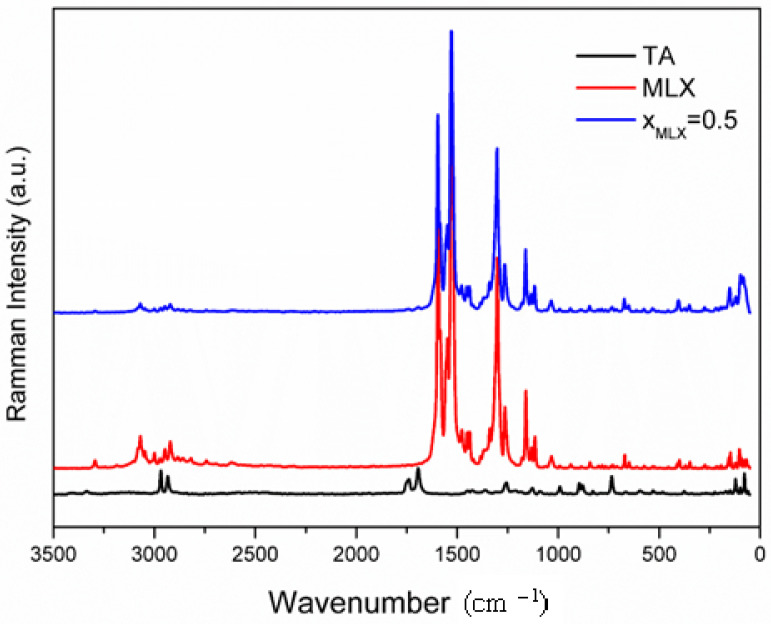
FT-Raman of pure meloxicam (red line), tartaric acid (black line) and their equimolar mixture (blue line).

FT-Raman spectra of pure meloxicam, tartaric acid and their equimolar mixture are given in Figure 6.

The Form I polymorph of meloxicam, the most stable one among the five known polymorphs, is emphasized with FT-Raman spectroscopy using the vibrational modes of C=O, C=C (1600–1400) cm^−1^, C–C (1300–1200) cm^−1^ and C-S (1200–800) cm^−1^ stretching [31,34,35]. Tartaric acid peaks are identified at 2968, 2934, 1739, 1693 and 1257 cm^−1^ [36]. 

Several shifts in band position were observed for the equimolar mixture of meloxicam—tartaric acid associated with co-crystal formation. A very interesting band intensity increase appeared for the co-crystal spectra so that laser power used for registering spectra was only 50 mW, ten times smaller than the 500 mW power used for registering the spectra of pure components due, probably, to the manifestation of the Raman resonance enhancement effect as a consequence of molecular interactions between meloxicam and tartaric acid, modifying electron and vibration energy levels. Also, in the region of crystal lattice vibrations, below 400 cm^−1^, important shifts in bands position and intensity appear, probing the formation of a co-crystal.

Similarly, in the case of the paracetamol–citric acid co-crystal, formation was followed by changes in the vibrational modes of amide and carboxylic acid groups; additionally, several new vibration bands appeared, which were not present in the raw materials [30].

### 3.3. PXRD Study

**Figure 7:**
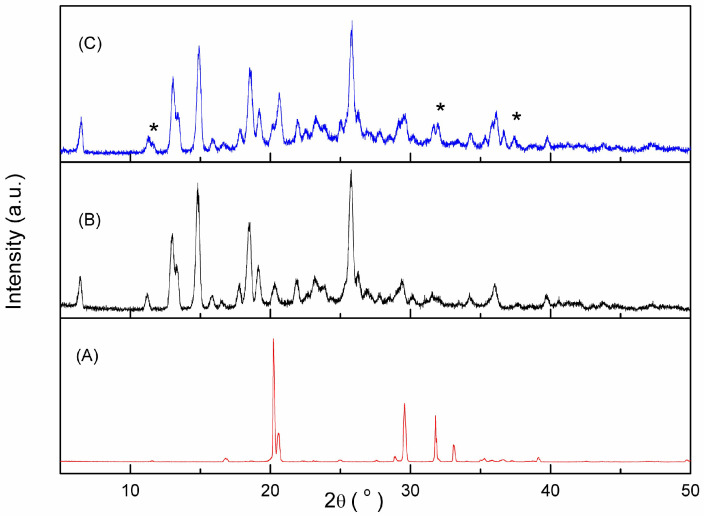
PXRD patterns of tartaric acid (A), meloxicam (B) and their co-crystal (C). (new diffraction peaks are marked with *).

The XRD patterns obtained for tartaric acid, meloxicam and their binary mixture with the molar fraction of meloxicam 0.5 are presented in Figure 7.

The characteristic diffraction peaks of meloxicam at 11.2, 13.0, 14.8, 17.8, 18.5, 19.2 and 25.8 degrees and those of tartaric acid at 16.8, 20.2, 20.6, 28.9, 29.6, 31.8, 31.9, 33.1 and 39.2 degrees could be seen in the diffraction pattern, and they are in accordance with the literature data [37]. In the PXRD diffractogram recorded for the binary mixture, new diffraction peaks appear at 2θ values of 11.62, 31.6 and 32.0 (marked with * in Figure 7), simultaneously with the disappearance of some existing lines in the tartaric acid diffractogram at 2θ values of 16.8, 28.9, 31.8, 31.9, 33.1 and 39.2. Therefore, the appearance of new diffraction lines, and the disappearance and displacement of others (compared to their positions observed for individual components) are considered evidence that further confirms the formation of the meloxicam–tartaric acid co-crystal. 

### 3.4. Solubility Tests

Solubility tests were performed for meloxicam and its co-crystal with tartaric acid by the saturation method. The determination of the meloxicam concentration was carried out by UV–Vis spectroscopy. The spectrum of meloxicam shows 2 peaks at 360 nm and 210 nm, while for tartaric acid, the UV–Vis spectrum shows a peak at 230 nm. To avoid unwanted interference in the determination of the concentration of meloxicam in the solution, the absorption band at 360 nm from the UV–Vis spectrum was chosen for analysis. The solubility of meloxicam was 8.62 μg·mL^−1^ in deionized water (see Table 2).

For meloxicam in the co-crystal, the determined solubility was 0.13 μg·mL^−1^, indicating a sharp decrease in its solubility in the co-crystal form compared to the pure, single, unmixed one. The observed aspect could be explained by the reported low solubility values of meloxicam in acidic and neutral aqueous media [9]. However, when the solubility tests were performed in a buffer solution of pH 7.4 (simulating the small intestine medium), the solubility of pure meloxicam was 108.6 μg·mL^−1^ and the solubility of meloxicam from the co-crystal mixture was 170.2 μg·mL^−1^, indicating an increase of 57% compared to that of the pure meloxicam. 

## 4. Conclusions

Meloxicam, tartaric acid and their mixtures were investigated by DSC, FTIR spectroscopy, PXRD, and solubility tests were performed. The physicochemical characteristics of the compounds obtained from the DSC data revealed a co-crystal at x_MLX_ = 0.5. Infrared spectroscopy data revealed the existence of hydrogen bonds between the co-crystal components, while the experimental results obtained by X-ray powder diffraction confirmed the formation of the co-crystal by the disappearance/appearance of peaks from the diffractogram of the co-crystal. FT-Raman spectroscopy correlated with the FTIR data, adding new information on the modification of the non-polar and crystal lattice bands vibration of pure APIs and their equimolar binary mixture. Co-crystal formation is followed by the conformational modification of the pure components due to hydrogen bonding and van der Waals interactions. Solubility tests in water have shown that the co-crystal product has a lower solubility, this being due to the acidic character of the other component—tartaric acid. Also, solubility tests in a buffer solution of pH 7.4 were performed. In this buffer, the solubility of meloxicam from the co-crystal was increased by 57%, compared to the pure meloxicam. Therefore, there is the possibility of opening a new research direction on the binary mixtures of meloxicam and new possible pharmaceutical formulations with absorption through the small intestine.

## Figures and Tables

**Table 1:** Decomposition temperature and mass loss for pure components and binary mixtures.

Compound	T_5%_ (K)	Δ*m* (%)	rm (%)
Meloxicam	546.65	77	23
Tartaric Acid	481.15	96	4
Meloxicam—Tartaric Acid (xMLX = 0.5)	474.4	82	18

*T*_5%_—initial decomposition temperature, Δ*m*—mass loss şi rm—residual mass at 600 °C.

**Table 2:** Meloxicam solubility from pure substance and its co-crystallization product in deionized water and in a buffer solution of pH 7.4.

Solubility Medium	Solubility (μg·mL^−1^)	
	Meloxicam	Co-Crystal
Deionized water	8.62	0.13
Buffer solution of pH 7.4	108.6	170.2

## Data Availability

Data are available upon request from the authors.
